# The first year of *RSC Chemical Biology* – a new journal was born

**DOI:** 10.1039/d1cb90001c

**Published:** 2021-02-01

**Authors:** Hiroaki Suga

**Affiliations:** Department of Chemistry, Graduate School of Science, The University of Tokyo 7-3-1 Hongo, Bunkyo-ku Tokyo 113-0033 Japan chembio-rsc@rsc.org

## Abstract

News from Hiroaki Suga, the Editorial Board Chair of *RSC Chemical Biology*.

## Graphical abstract

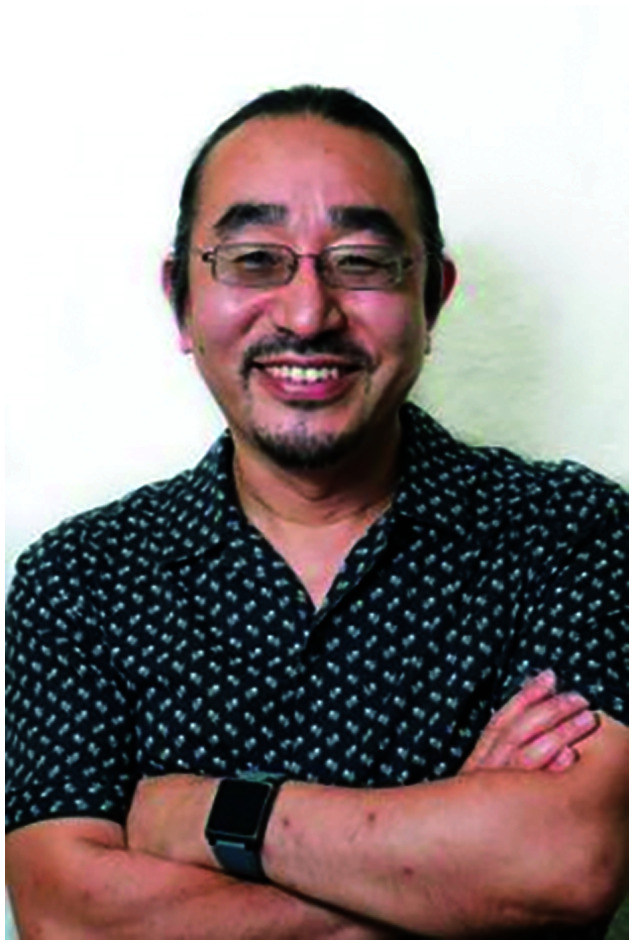


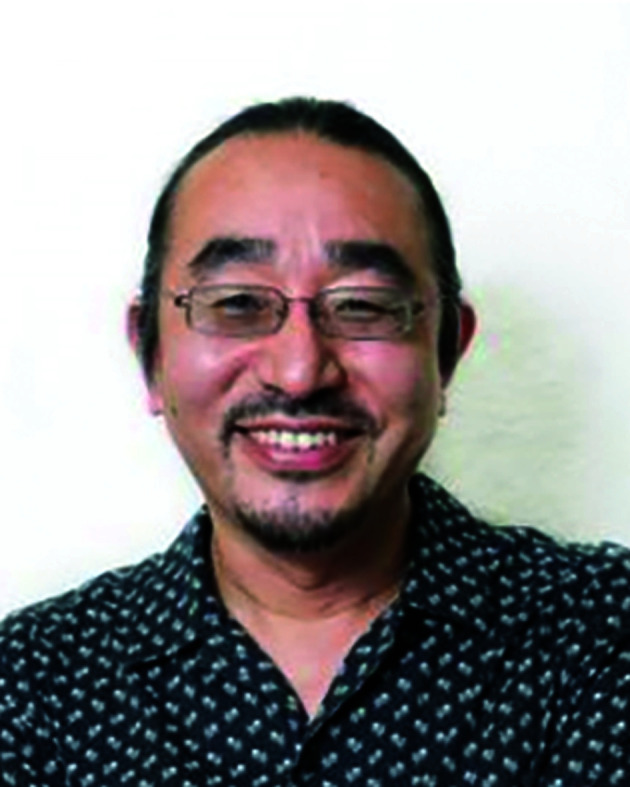
 **Hiroaki Suga**

It was during the 2019 IUPAC meeting in Paris when I first discussed launching a new journal with a focus on the field of chemical biology with the Royal Society of Chemistry (RSC). Despite there being several excellent journals in the chemical biology field already available at the time, I was excited about this new launch since the field is continuously expanding and there was definitely space for a new journal. As a consequence of this encounter in Paris, and after exchanging many e-mails with the RSC Editorial Office, I was invited to become the Editorial Board Chair of *RSC Chemical Biology* and help build the team of Associate Editors and Editorial Board members. I agreed, and began working closely together with the RSC Editorial Office to build our expert Editorial Board to span the diverse gender, geography, and expertise of chemical biology researchers, commissioning high-quality papers and launching our first issue of *RSC Chemical Biology* in April 2020.

*RSC Chemical Biology* is undeniably inimitable in several ways.

First of all, it is a gold Open Access journal. This is especially important for the community as there had been no specialized open access journal in this area until the launch of *RSC Chemical Biology*. We believe by making research freely available to anyone who needs it, gold open access publishing allows wider knowledge dissemination, removes reading restrictions, and offers more opportunities for researchers to improve the visibility of their work and build a strong reputation. Because of this, science can progress faster. This is good for everyone.

Second, we offer our authors a Transparent Peer-Review (TPR) option. If authors choose this option the full history of peer-review is published alongside their manuscript. We launched this initiative, further demonstrating our commitment to open science, to combat bias in publishing and to openly demonstrate that our editors and reviewers are both held accountable and acknowledged (I have to highlight here that the reviewers remain anonymous unless they decide to sign their report). *RSC Chemical Biology* was the first journal at the RSC to launch TPR and indeed, with half of our submitting authors opting for this option, we are pleased to be offering and meeting the interest of our community in this way. I expect that this number will increase when the benefits of TPR become more widely recognized and established among scientists.

Our 2020 volume includes review, research, communication and opinion articles; in terms of modalities of molecules, research including small molecules, peptides/peptidomimetics, nucleic acids, natural products/secondary metabolites, and proteins are all featured; and in terms of topics in chemical biology, we published research on drug discovery, biosynthesis, enzyme and cellular mechanisms, probes/tools, and more. Most importantly, at least to me, all research published involved significant components of chemistry as an RSC journal. Some stand-out examples for me include:

• “Harnessing the PD-L1 interface peptide for positron emission tomography imaging of the PD-1 immune checkpoint” by Hu *et al.*, DOI: 10.1039/D0CB00070A.

The authors of this paper have developed an impressive PET imaging tool for the most famous immune check point mediated by the PD-1 and PD-L1 interaction, demonstrating not only cell culture and *ex vivo* detection but also *in vivo* detection in mice.

• “A thorough analysis and categorization of bacterial interrupted adenylation domains, including previously unidentified families” by Lundy *et al.*, DOI: 10.1039/D0CB00092B.

The families of interrupted A domains and types of M domains in the nonribosomal peptide class of natural products have been categorized. It has illuminated patterns and insights on how to harness them for engineering studies in the future.

• “The chemical biology of coronavirus host–cell interactions” by Datta *et al.*, DOI: 10.1039/D0CB00197J.

This timely review comprehensively summarizes the biological events linked to the coronavirus outbreak.

 

As we welcome you to the opening issue of our 2021 volume, we want to share our focus for 2021 to continue increasing the excellent quality and quantity of the manuscripts published in *RSC Chemical Biology*, and welcome you as our growing community.

Hiroaki Suga

Professor, University of Tokyo.

Editorial Board Chair, *RSC Chemical Biology*

## Supplementary Material

